# Recurrence Patterns in Breast Cancer: A Single-Center Retrospective Analysis

**DOI:** 10.3390/jcm14228243

**Published:** 2025-11-20

**Authors:** Cristina Marinela Oprean, Teodora Hoinoiu, Larisa Maria Badau, Radu Vidra, Tiberiu Dragomir, Gabriel-Mugurel Dragomir, Daniel Piț, Alexandru Catalin Motofelea, Nadica Motofelea, Alis Dema, Daciana Grujic

**Affiliations:** 1 ANAPATMOL Research Center, “Victor Babes” University of Medicine and Pharmacy, Eftimie Murgu Square No. 2, 300041 Timisoara, Romania; cristina.oprean@umft.ro (C.M.O.); dema.alis@umft.ro (A.D.); 2 Department of Oncology, ONCOHELP Hospital Timisoara, Ciprian Porumbescu Street No. 59, 300239 Timisoara, Romania; larisa.badau@umft.ro; 3 Department of Oncology, ONCOMED Outpatient Unit, Ciprian Porumbescu Street No. 59, 300239 Timisoara, Romania; 4 Department of Clinical Skills, “Victor Babes” University of Medicine and Pharmacy, Eftimie Murgu Square No. 2, 300041 Timisoara, Romania; daniel.pit@umft.ro (D.P.); nadica.motofelea@umft.ro (N.M.); 5 Center for Advanced Research in Cardiovascular Pathology and Hemostaseology, “Victor Babes” University of Medicine and Pharmacy, 300041 Timisoara, Romania; 6 “Pius Brinzeu” County Emergency Hospital, 300723 Timisoara, Romania; grujic.daciana@umft.ro; 7 Hygiene Discipline, “Victor Babes” University of Medicine and Pharmacy, Eftimie Murgu Square No. 2, 300041 Timisoara, Romania; 8 MEDFUTURE Biomedical Research Institute, Department of Personalized Medicine and Rare Diseases, Iuliu Hațieganu University of Medicine and Pharmacy, 400347 Cluj-Napoca, Romania; radu.vidra@irgh.ro; 9 UBBMed & The International Institute for the Advanced Studies of Psychotherapy and Applied Mental Health, Babes-Bolyai University, 400347 Cluj-Napoca, Romania; 10 Department V Internal Medicine, The Discipline of Medical Semiology II, “Victor Babes” University of Medicine and Pharmacy, Eftimie Murgu Square No. 2, 300041 Timisoara, Romania; dragomir.tiberiu@umft.ro; 11 Department of Teaching Training, Polytechnical University of Timisoara, 300223 Timisoara, Romania; mugur.dragomir@upt.ro; 12 Doctoral School, “Victor Babes’’ University of Medicine and Pharmacy, Eftimie Murgu Square No. 2, 300041 Timisoara, Romania; alexandru.motofelea@umft.ro; 13 Center for Molecular Research in Nephrology and Vascular Disease, Faculty of Medicine, “Victor Babes” University of Medicine and Pharmacy, 300041 Timisoara, Romania; 14 Department of Obstetrics and Gynecology, “Victor Babes” University of Medicine and Pharmacy Timisoara, Eftimie Murgu Square No. 2, 300041 Timisoara, Romania; 15 Discipline of Morphopathology, “Victor Babes” University of Medicine and Pharmacy, Eftimie Murgu Square No. 2, 300041 Timisoara, Romania; 16 Department of Plastic and Reconstructive Surgery, “Victor Babes” University of Medicine and Pharmacy, Eftimie Murgu Square No. 2, 300041 Timisoara, Romania

**Keywords:** breast cancer recurrence, molecular subtype, postmenopausal status, socioeconomic disparities, breast cancer treatment, retrospective analysis

## Abstract

**Background:** Breast cancer mortality and long-term survival are influenced by the unpredictability of recurrences, which cause significant diagnostic and therapeutic challenges for oncology teams. The risk of local and distant recurrence is higher in advanced stages and in the first two years following initial treatment. Accurate staging and continuous monitoring of recurrence are crucial for effective therapy planning. Indicators of recurrence, such as luminal subtype, disease stage, age, and treatment choice, can provide new knowledge and improve patient disease-free and overall survival rates. **Methods:** We conducted a retrospective cohort study of patients with stage I-III invasive breast cancer at a regional-based institution. The study population consisted of 98 patients with distant and locoregional recurrences from a large cohort of 744 patients diagnosed and treated at our institution between 2007 and 2024. Data on previous treatment for breast cancer, disease stage, molecular subtype, initial size and location of the tumor in the breast, lymph node status, living environment, and type of recurrence were recorded retrospectively. **Results:** The recurrence patterns in 98 patients included local recurrence in 25 (25.5%), distant recurrence in 70 (71.4%), and both local and distant recurrence in three (3.1%). Our study showed that patients diagnosed with stage II (40.8%) or stage III (55.1%) breast cancer, as well as those with the luminal B subtype (43.87%), were more likely to experience recurrence. The majority of patients affected by recurrent disease were postmenopausal women aged between 51 and 70 years (32 cases aged 51–60 years and 34 cases aged 61–70 years). Tumors measuring between 2 and 5 cm were more likely to produce distant single-organ recurrence (26 cases). More cases were associated with urban areas (77 cases). **Conclusions:** In menopausal women, most causes of local breast cancer recurrence are related to advanced stage at diagnosis and luminal B subtype. Patient age, primary tumor location in the CSE, and previous adjuvant treatment with aromatase inhibitors may affect the risk of recurrence. Comprehensive studies on recurrence in postmenopausal women can provide a more precise understanding of the extent of disease in such patients.

## 1. Introduction

Trends in mortality show significant differences between Eastern and Western European countries [1,2]. Currently, there are no comprehensive national data on the 5-year survival rate after breast cancer diagnosis in Romania. The only available estimate, 77.29%, is based on data from the northwestern regional counties and likely overestimates the national average [3]. Moreover, compared to other countries, the survival of female patients with BC in Romania is lower, considering that several European countries, such as Sweden, Finland, Norway, Switzerland, the Netherlands, and Italy, have reported a 5-year survival rate of over 83% (measured between 2000 and 2002) [4].

BC recurrence is the principal cause of BC-related death [5]. Recently, researchers have attempted to predict breast cancer recurrence patterns. A set of different factors, such as breast cancer (BC) stage, tumor biology, molecular subtype, diagnostic procedures, adherence and access to treatment standards, treatment toxicity, pre-existing chronic conditions, and socioeconomic disparities, influence BC survival [1,2]. This includes studies on various BC subtypes associated with distinct patterns of local and metastatic spread [5,6]. A differential recurrence pattern has been suggested for different BC subtypes. Estrogen Receptor (ER)-negative BC is associated with a higher risk of recurrence during the initial 5 years after diagnosis than ER-positive BC [7].

The primary prognostic factors for BC outcomes are tumor characteristics and patient age at the time of diagnosis. A tumor size larger than 2 cm with axillary nodal involvement, hormone receptor status (harmful estrogen and progesterone receptors), and high-grade vascular invasion have been shown to increase the risk of locoregional recurrence and death after BC diagnosis [8,9].

The risk of local and distant recurrence is higher in advanced stages and in the first two years following initial treatment. Other studies have described prolonged intervals (5–8 years) for the risk of recurrence after systemic treatment [10]. Recurrence rates may vary depending on prognostic factors, tumor biology, and molecular subtypes of the disease. Thus, type HER2-positive and triple-negative breast cancers relapse two to three times more often than luminal A or B.

After breast-conserving surgery (BCS), the conserved breast (or the axilla if it has not been effectively treated) is a prevalent site of local recurrence. The risk of recurrence in a conserved breast can be substantial, even in node-negative disease confirmed by axillary clearance, and can be significantly reduced by radiotherapy [8].

A dynamic model was designed to predict the risk of death using a history of cancer recurrence in the French E3N cohort [11]. According to this study, postmenopausal status was not a significant risk factor for mortality. However, weight, which is associated with a higher risk of postmenopausal BC, was associated with a higher risk of BC recurrence and death. Postmenopausal women with less than 5 years of cumulative menopausal hormonal treatment (MHT) were included in the low-risk profile group. In contrast, postmenopausal women not receiving MHT were included in the high-risk profile group.

Cancer relapse in postmenopausal women has not yet been studied. Furthermore, there is limited prognostic information to identify postmenopausal patients with BC who are at risk of late recurrence after adjuvant or neoadjuvant systemic therapies.

Our study aimed to provide an overview of our current findings regarding BC recurrence by molecular subtype, stage at diagnosis, lymph node status, tumor size, initial tumor location in the breast, age, living environment, and treatment-related interventions. The selected patient population (East European Caucasian postmenopausal women) is currently under-studied. Our study results provide further insights into the prevention and management of breast cancer recurrence and improvement of patient disease-free survival. However, owing to sample size limitations in this subgroup, these results should be interpreted with caution and validated in larger samples.

## 2. Materials and Methods

This retrospective, observational, unicentric study was conducted in a general oncology specialized outpatient unit at the West Oncology Center, Romania.

Four types of recurrent events were considered in our study: local recurrence, single-organ distant recurrence, multi-organ distant recurrence, and a composite of local and distant recurrence.

### 2.1. Study Design and Population

We analyzed the patient registry of our Oncology-specialized Outpatient Unit for this population-based cohort study. This registry contains prospectively recorded patient-, tumor-, and treatment-related characteristics of all newly diagnosed patients with BC. We included 744 patients diagnosed between May 2007 and December 2024 who were treated with either breast-conserving therapy or mastectomy, comprising 736 (98.9%) postmenopausal women and eight male patients (1.1%). The disease stage was defined according to the 4th edition of the Tumor, Nodes, Metastasis (TNM) manual [12].

If pathological data on tumor size and lymph node status (pT and pN) were unavailable, clinical data (cT and cN) were used. Immunohistochemical characterization and molecular subtype data were collected. Patients with incomplete data on history, treatment, and follow-up were excluded from the study.

Patients received different treatment combinations for primary BC, including surgery (*n* = 91), chemotherapy (*n* = 98), hormone therapy (*n* = 82), and radiotherapy (*n* = 70).

The Institutional Review Board (IRB) of the ONCOMED Outpatient Unit approved this retrospective study in July 2024 [13]. Retrospective data were collected and reviewed in compliance with the ethical standards set out by the IRB and the Declaration of Helsinki. For this retrospective study, the Institutional Review Board did not require patient consent to review the medical records. As we considered the present study an ancillary study to further explore the characteristics of recurrence in BC, additional approval from the IRB was not deemed necessary.

### 2.2. Data Collection

Data were collected with the support of the ONCOMED Outpatient Unit. The population registry included 98 eligible patients with locoregional recurrence who were diagnosed and treated at our institution between 2007 and 2024. Data on initial treatment for breast cancer, initial disease stage, molecular subtype, site of the primary tumor in the breast, lymph node status, tumor dimension, and type of recurrence were recorded retrospectively. All personal data of the study population were anonymized, and the IRB approved the study.

We considered demographic data, including age (defined as the age at the time of diagnosis) and living environment (rural or urban).

The clinical data collected included the disease stage at the time of diagnosis. Clinical data recorded in oncology outpatient charts and surgical medical letters were also used as documentation sources. Clinically and pathologically, TNM classifications were used to establish the disease stage. Pathological TNM staging was used for patients who underwent surgery. Clinical TNM staging was used for the remaining patients. Depending on the year of diagnosis, we used the 5th, 6th, and 7th editions of the AJCC TNM classification [14,15].

The molecular subtypes of invasive tumors were classified according to the 2017 St Gallen Consensus, based on estrogen receptor (ER), Progesterone Receptor (PR), cell proliferation-associated antigen (Ki-67), and HER2 status. According to this classification, patients were divided into five subgroups: luminal A, luminal B, luminal HER-positive, non-luminal HER2-positive, and triple-negative breast cancer [16].

These parameters were obtained from immunohistochemical (IHC) examination of the breast cancer tissue samples. The tissue samples were examined in three local laboratories based in Timisoara, Romania. All laboratories used either manual or automated platforms for IHC analysis. ER and/or PR were considered positive when the values were >1%. The cut-off value for KI-67 to distinguish between luminal A and luminal B tumors was set at 20%. To distinguish between HER2-positive and HER2-negative tumors, pathologists performed IHC and in situ hybridization tests using the ASCO/CAP guidelines that were valid at the time of examination.

### 2.3. Statistical Analysis

All statistical analyses were conducted using the Statistical Package for the Social Sciences (SPSS) version 22 (IBM Corp., Armonk, NY, USA). Descriptive statistics were used to summarize the demographic and clinical characteristics of the study cohort, including measures of central tendency (mean, median), dispersion (standard deviation), and proportions (percentages) for categorical variables.

The primary outcomes of interest were recurrence patterns (local, distant, single-organ, and multiorgan) in relation to clinicopathological variables such as tumor stage, molecular subtype, primary tumor location, treatment modality, and tumor size. Comparisons between categorical variables were made using the Pearson Chi-square test or Fisher’s exact test (where appropriate) to assess statistical significance across groups. Statistical significance was set at *p* < 0.05. Survival outcomes were estimated using the Kaplan–Meier method, and survival curves were generated to illustrate the time-to-event data. Although not reported in this summary, differences in survival curves were compared using the log-rank test where applicable.

Cross-tabulations were used alongside chi-squared testing to assess the associations between recurrence patterns and explanatory variables (molecular subtype, tumor size, quadrant location, and adjuvant treatment). Owing to the retrospective nature and limited event counts in some strata (distant recurrences), multivariate analyses such as logistic regression or Cox proportional hazards models were not applied but may be considered in future larger-scale studies.

Due to the high proportion of missing values in some variables (notably recurrence type), sensitivity analyses were not performed, and the results should be interpreted with caution. Missing data were reported explicitly, but no imputation methods were used for them.

## 3. Results

In a cohort of 744 patients, the mean age at diagnosis was 62.4 ± 9.2 years (range, 33.0–87.0 years), and the gender distribution was predominantly female, with 736 (98.9%) females compared with 8 (1.1%) males. Most patients were urban residents (597 [80.2%]), and 147 (19.8%) were rural residents.

The distribution of cancer stages at diagnosis was heterogeneous: 93 patients (12.5%) had stage I disease, 301 (40.5%) had stage II disease, 238 (32.0%) had stage III disease, and 112 (15.1%) had stage-IV disease.

Recurrence data were incomplete, with 614 cases lacking information on recurrence. Among the 130 patients with available data, 25 (19.2%) had local recurrence, 3 (2.3%) had distant recurrence, 35 (26.9%) had multiorgan recurrence, and 67 (51.5%) had single organ recurrence (Table 1).

**Figure 1:**
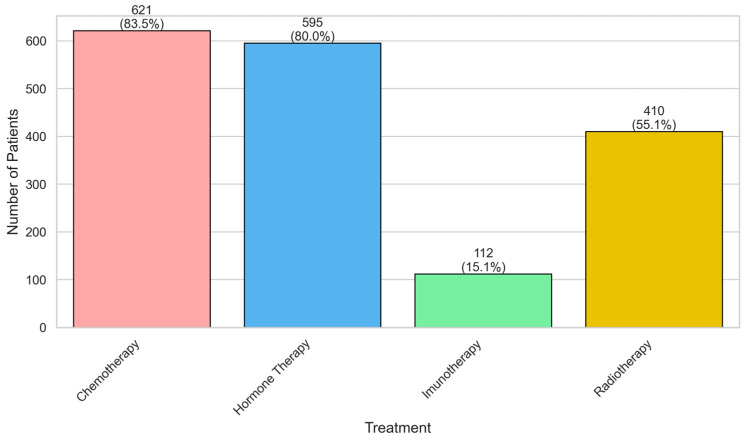
Distribution of treatments among patients with breast cancer.

Chemotherapy 621 (83.5%) and hormone therapy 595 (80.0%) were the most frequently administered treatments, followed by radiotherapy 410 (55.1%) and immunotherapy 112 (15.1%), showing the predominant use of systemic therapies and limited adoption of immunotherapy (Figure 1).

Among the 130 patients with available recurrence data, recurrence patterns varied according to disease stage. In stage I disease, 5 (3.8%) patients developed recurrence, predominantly single-organ 4 (6.0%) and one multiorgan 1 (2.9%). Stage II disease accounted for 27 (20.8%) and 20 (15.4%) recurrences in stages IIA and stage IIB, respectively, with local and single-organ events predominating. Stage III subgroups showed higher recurrence frequencies: 23 (17.7%) in stage IIIA, 32 (24.6%) in stage IIIB, and 11 (8.5%) in stage IIIC, most commonly multi-organ or single-organ. In contrast, only eight (6.2%) patients with stage IV disease experienced recurrence, mainly single- or multi-organ events. Advanced stages (III–IV) were associated with higher and more complex recurrence patterns than early stages (I–II). (Table 2).

**Figure 2:**
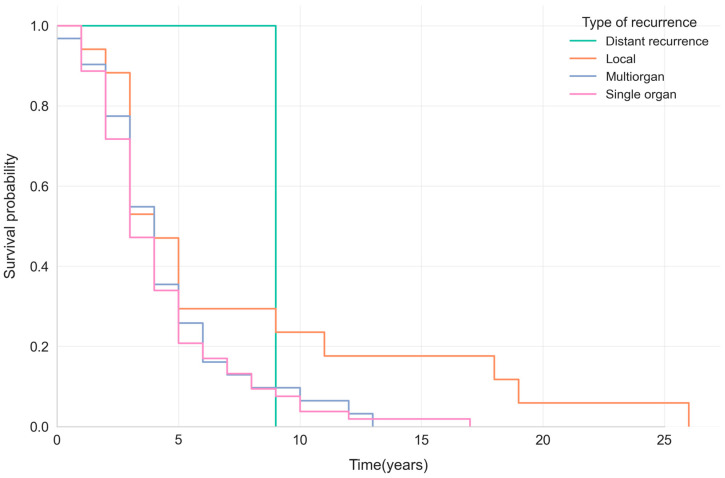
Kaplan–Meier survival curves showing survival probabilities by recurrence pattern (distant, local, multiorgan, or single-organ) in patients with breast cancer.

Among the 130 patients with available recurrence data, the distribution of recurrence types varied across disease stages. Of these, 3 (2.3%) patients experienced distant recurrence, 25 (19.2%) local recurrence, 35 (26.9%) multiorgan recurrence, and 67 (51.5%) single-organ recurrence. The *p*-value for differences across stages was 0.3011, indicating no statistically significant variation in recurrence patterns by stage (Figure 2).

**Figure 3:**
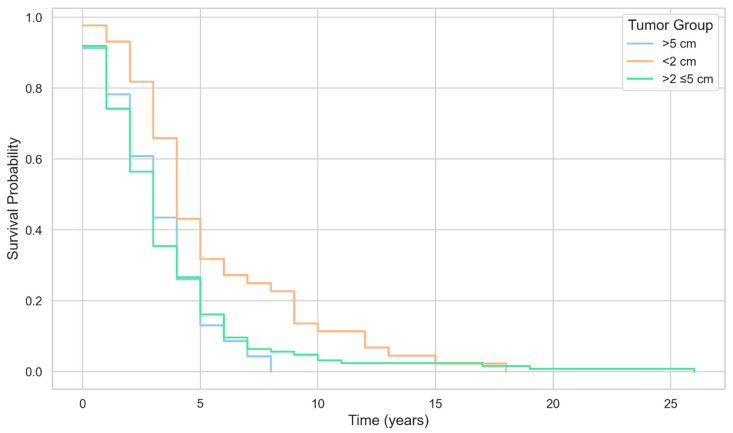
Kaplan–Meier survival curves by tumor size group among patients with breast cancer.

The Kaplan–Meier curves showed that survival after breast cancer recurrence is generally poor, with a median survival ranging from 3 to 4 years across recurrence types. Patients with local recurrence showed a slightly higher 5-year survival probability (~29%) than those with systemic (single- or multiorgan) recurrence (~20–26%) (Figure 3).

Recurrence type was available for 130 of the 744 patients, with missing information in 614 patients. Of those with data, 38 of 269 had subtype A, 63 of 307 had subtype B, 14 of 79 had luminal HER2-positive disease, 3 of 31 had non-luminal HER2-positive disease, and 12 of 58 had triple-negative breast cancer (TNBC). The distribution of recurrence patterns differed significantly among the subtypes (*p* = 0.0351).

In subtype A, distant, local, multiorgan, and single-organ recurrences occurred in one (2.6%), eight (21.1%), 13 (34.2%), and 16 (42.1%) patients. Among subtype B, 1 (1.6%) had distant, 9 (14.3%) local, 19 (30.2%) multiorgan, and 34 (54.0%) single-organ recurrences. In luminal HER2-positive cases, there were no distant recurrences; 4 (28.6%) were local, 1 (7.1%) multiorgan, and 9 (64.3%) single-organ. Among the three non-luminal HER2-positive patients, one (33.3%) had a distant recurrence, and two (66.7%) had single-organ recurrence. Of the 12 triple-negative cases, 4 (33.3%) were local, two (16.7%) were multi-organ, and six (50.0%) were single-organ. Recurrence predominated across most subtypes, except in non-luminal HER2-positive disease, where distant recurrence accounted for one-third of events (Table 3).

In a subset of 130 patients with documented recurrence, immunotherapy was administered to 28 (21.5%) patients and withheld from 102 (78.5%) patients. Among patients with distant recurrence (*n* = 3), two (66.7%) had not received immunotherapy and one (33.3%) had received immunotherapy. Local recurrence (*n* = 25) occurred in 18 (72.0%) patients without immunotherapy and seven (28.0%) with it; multiorgan recurrence (*n* = 35) occurred in 32 (91.4%) untreated and three (8.6%) treated patients; and single-organ recurrence (*n* = 67) occurred in 50 (74.6%) untreated and 17 (25.4%) treated patients (*p* = 0.177).

Chemotherapy was administered to 117 (90.0%) and omitted in 13 (10.0%) patients. All distant recurrences occurred in individuals who received chemotherapy. Of the patients with local recurrence, 24 (96.0%) were treated and 1 (4.0%) was untreated; of those with multiorgan recurrence, 29 (82.9%) were treated and 6 (17.1%) were untreated; and those with single-organ recurrence, 61 (91.0%) were treated and 6 (9.0%) were untreated (*p* = 0.334).

Radiotherapy was delivered to 91 (70.0%) and withheld from 39 (30.0%) patients. Distant recurrence involved two (66.7%) irradiated and one (33.3%) non-irradiated patient; local recurrence involved 19 (76.0%) irradiated and 6 (24.0%) non-irradiated patients; multiorgan recurrence involved 23 (65.7%) irradiated and 12 (34.3%) non-irradiated patients; and single-organ recurrence involved 47 (70.1%) irradiated and 20 (29.9%) non-irradiated patients (*p* = 0.861).

Hormone therapy was administered to 109 (83.8%) patients and omitted in 21 (16.2%) patients. Two (66.7%) of the three distant recurrences occurred in hormone-treated patients. Among those with local recurrence, 20 (80.0%) were treated and five (20.0%) were untreated. Multiorgan recurrence occurred in 31 (88.6%) treated and four (11.4%) untreated patients, and single-organ recurrence occurred in 56 (83.6%) treated and 11 (16.4%) untreated patients (*p* = 0.681).

None of the four treatment modalities showed a statistically significant association with recurrence patterns, indicating comparable distributions of distant, local, multiorgan, and single-organ relapse, regardless of exposure to immunotherapy, chemotherapy, radiotherapy, or hormone therapy (Table 4).

Among patients with distant recurrence (*n* = 3), two (66.7%) had upper outer quadrant (UOQ) and one (33.3%) had upper inner quadrant (UIQ) tumors. In local recurrences (*n* = 25), UOQ tumors predominated (16, 64.0%), followed by CS and UIQ (3 each, 12.0%), LIQ (2, 8.0%), and LOQ (1, 4.0%) tumors. Multiorgan recurrences (*n* = 35) most often originated from LOQ (16, 45.7%), CS (9, 25.7%), and UIQ (6, 17.1%) primaries. Among single-organ recurrences (*n* = 67), UOQ was the most frequent (31, 46.3%), followed by UIQ (13, 19.4%), CS (12, 17.9%), LOQ (8, 11.9%), and LIQ (3, 4.5%) recurrences. Overall, half of all recurrences (65, 50.0%) arose from UOQ tumors, with CS, UIQ, LOQ, and LIQ accounting for 24 (18.5%), 23 (17.7%), 11 (8.5%), and 7 (5.4%) events, respectively. No recurrences originated from the bilateral primary sites. Recurrence patterns did not differ significantly according to tumor location (*p* > 0.05) (Table 5).

Among patients with available recurrence-type data (*n* = 123), single-organ recurrence was the predominant pattern in every tumor-size group 12/25 (48.0%) for <2 cm, 45/87 (51.7%) for >2–≤5 cm, and 7/11 (63.6%) for >5 cm—yielding 64/123 (52.0%).

Multiorgan recurrence accounted for 9/25 (36.0%), 21/87 (24.1%), and 3/11 (27.3%) across the three strata (33/123 (26.8%). Local recurrence comprised 3/25 (12.0%), 20/87 (23.0%), and 1/11 (9.1%) (24/123 [19.5%]. Distant recurrence was rare (≤4.0% in any stratum; 2/123 [1.6%].

The Pearson’s χ^2^ *p* = 0.6191 indicates no statistically significant difference in the distribution of recurrence types across tumor-dimension groups (Table 6).

Among the 130 patients with documented recurrence, treatment exposure was as follows: radiotherapy, 91 (70.0%); chemotherapy, 117 (90.0%); immunotherapy, 28 (21.5%); and hormone therapy, 109 (83.8%). Across modalities, the recurrence pattern was broadly similar: single-organ events were most frequent (64 [52.0%]) and distant events were rare 2 (1.6%). The distribution of recurrence types did not differ according to radiotherapy (*p* = 0.861), chemotherapy (*p* = 0.334), immunotherapy (*p* = 0.177), or hormone therapy (*p* = 0.681). Numerically lower local and multiorgan events were observed in patients who received immunotherapy; however, this difference was not significant (Table 7).

## 4. Discussion

Our study aimed to identify the profiles of local and distant recurrence in Caucasian postmenopausal women treated at our institution.

Despite advances in early detection and optimization of treatments for BC, approximately 30% of patients with early-stage BC experience recurrent disease [17].

Recurrent disease was associated with traditional histopathological parameters, including lymph node status, histological grade, and tumor size. The timing of recurrence was correlated with the hormone receptor status, independent of the histological type. The highest risks were observed among ER-PR—patients within two years of surgery [17].

Our study showed that the highest percentage of recurrence was observed in patients initially diagnosed with stage III (55.1%), followed by those diagnosed with stage II (40.8%). Indeed, inferior access or use of breast cancer screening can lead to a postponement in the detection of a breast tumor until it becomes symptomatic, which increases the likelihood that it will be in an advanced stage at the time of diagnosis.

A study on BC subtypes and the risk of local and regional relapse conducted by Voduc concluded that larger tumors were associated with a higher risk of recurrence in the chest wall [18]. In contrast, in the postmenopausal women population that we studied, we found that medium-sized tumors—2–5 cm led to a higher number of BC recurrences.

The impact of molecular subtypes on prognosis and recurrence patterns can also be predicted. We analyzed the molecular subtypes associated with BC recurrence in our study population. We conclude that breast cancer non-luminal HER positive manifested in fewer cases of recurrence: two cases of single-organ distant recurrence and one case of local and distant recurrence, which can be associated with a lower risk of local or regional recurrence in postmenopausal women. The most frequent recurrence we observed was among the Luminal B subtype, and within this molecular subtype, single distant recurrence was predominant.

Lymph node status is a critical prognostic factor [5,6]. Pan et al. (2017) [19] and the Early Breast Cancer Trialists’ Collaborative Group (EBCTCG, 2024) reported higher recurrence risks for N2/N3 than for N1/N0 over 5–20 years (hazard ratios: 2.5–3.0 for N2, >3.0 for N3) [15,16]. This study’s finding that N-staging is not significantly associated with recurrence (*p* > 0.05) contrasts with these data, likely due to the limited number of recurrence events (*n* = 130) and missing data (614/744). The trend of N1/N2 favoring multiorgan recurrence aligns with that of Wang et al. (2018), who noted increased multiorgan metastases for N1 (HR 1.8) and N2 (HR 2.5) [18]. The lower multiorgan recurrence in N3 may reflect aggressive treatments (90.0% chemotherapy, 83.8% hormone therapy), which mitigated widespread metastasis. The lack of association with single-organ recurrence is consistent with the findings of Xu X et al. (2020), who found that single-organ metastases are driven by tumor biology [20].

The well-studied disparities in BC, including socioeconomic disparities, show that the lack of economic resources combined with the lack of health literacy complicates the diagnosis and treatment of early-stage BC in patients originating from rural environments. Even if the urban environment can trigger more BC recurrences, we assume that the population in rural areas is underrepresented and, as such, underdiagnosed [21]. In this regard, we performed an additional correlation analysis to determine the value of both rural and urban environments in relation to the number of BC recurrence cases. Our study results showed that most patients came from urban environments and experienced all types of recurrence. The percentage of patients from rural environments enrolled in this study was almost four times lower than that of urban patients. This significant difference may have introduced bias in our results.

The analysis of how the quadrant localization of the primary tumor in the breast influences the recurrence rate in our study showed that localization in the upper outer quadrant is associated with a higher rate of recurrence, including both local and single-organ distant recurrence. This finding is consistent with that of Drew et al. [22]. In this regard, our study results are in concordance with other studies [18,20,22]; the upper outer quadrant localization of the primary BC tumor was found to be correlated with local recurrence in 16 analyzed cases (46%) and with single-organ distant recurrence in 23 cases (64%). Among all patients who experienced recurrence, 51% had an initial tumor in the upper outer quadrant.

Studies have reported that after mastectomy without reconstruction, cancer recurs more often in the cutaneous or subcutaneous tissue of the mastectomy flap [23], and recurrence in the deeper tissues of the chest wall is infrequent. Traditionally, both sites have been grouped under the category of “locoregional recurrence,” which also includes regional recurrence in the axilla.

In our study, the results showed that distinct risk factors for recurrence exist in patients with breast cancer undergoing mastectomy versus those choosing breast-conserving therapy. Our findings are consistent with those of Ahmad et al. [5]. We observed recurrence in 91 cases (91.8%) patients who underwent surgical treatment, that is, mastectomy, versus only seven (7.1%) patients who underwent breast-conserving therapy. These results may not be relevant because of the large difference in the percentage of patients treated mostly with radical mastectomy. Unfortunately, this also reflects the daily surgical practice in the western part of Romania over the past decade, with a predominance of radical surgery for breast cancer.

After the primary BC tumor is removed by lumpectomy or mastectomy, some patients should receive full-dose radiotherapy to the chest wall and regional lymph nodes to reduce the risk of local BC recurrence. Neoadjuvant and adjuvant chemotherapy are administered to delay the possible onset of distant organic recurrence. The choice of systemic treatment should consider the clinical and molecular characteristics of the tumor, as outlined in the guidelines.

According to a meta-analysis of 10,801 patients from 17 randomized trials published by the Early Breast Cancer Trialists’ Collaborative Group, radiation therapy reduces the recurrence rate by half and the death rate by approximately one-sixth in patients who have undergone breast-conserving therapy [24]. Consistent with these results, a differential response to radiotherapy was observed in our study: distant recurrences, both single and multi-organ, were observed more frequently in patients who did not receive radiotherapy in the initial stages.

Data from various scientific studies suggest the usefulness of aromatase inhibitors in preventing early-stage distant metastases in postmenopausal women. Therefore, it has been proposed that initial therapy involving the adjuvant use of aromatase inhibitors should be the first choice for these patients [25,26]. Most patients who received adjuvant hormone therapy (HT) with aromatase inhibitors (AI) were clinically staged II or III at diagnosis or exhibited aggressive pathological features (e.g., Luminal B subtype). None of the treatment modalities significantly influenced recurrence patterns (*p* = 0.177–0.861). Data from various studies indicate that aromatase inhibitors are beneficial in preventing early-stage distant metastasis in postmenopausal women. Consequently, starting with adjuvant aromatase inhibitors is recommended, especially for patients with more aggressive disease due to higher stage (e.g., stage III) or tumor biology (e.g., high Ki-67 in luminal B subtypes) [26,27,28]. The trend toward lower multiorgan recurrence with immunotherapy (8.6%) suggests its potential efficacy in aggressive subtypes [8,29]. The high recurrence rate after mastectomy (91.8%) may reflect a selection bias for advanced disease [1,19,25]. The lack of impact from radiotherapy contrasts with the EBCTCG (2011), which reported a 50% reduction in recurrence rates [17,30,31,32].

This study had some limitations. First, it was a retrospective analysis with a small sample size, limited to postmenopausal Caucasian women. Second, the choice of treatment may lead to differences in outcomes, especially in patients with HER-2-positive disease who receive targeted anti-HER2 agents. Third, our population, comprising 98 patients with BC recurrence, showed that the luminal B subtype accounted for a much higher proportion than that reported previously. Additionally, there may have been potential selection/information and confounding bias.

This study provides an important basis for future investigations into the mechanisms underlying breast cancer recurrence in postmenopausal women. By identifying key factors such as advanced disease stage at diagnosis, luminal B molecular subtype, upper outer quadrant localization, mastectomy, and treatment with aromatase inhibitors, this study underscores the need for larger and more comprehensive studies to confirm these associations. Future research can build on these findings to refine risk prediction models, improve the early detection of recurrence, and develop individualized prevention and management strategies aimed at prolonging disease-free survival in this patient population.

## 5. Conclusions

Breast cancer recurrence is a poor prognostic indicator. The cause(s) of breast cancer recurrence and possible strategies for its prevention have not yet been defined. There is an urgent need to establish correlations between BC recurrence and distinct clinical, biological, and individual factors that may contribute to its onset of BC recurrence.

We concluded that the following factors may drive breast cancer recurrence in postmenopausal women: advanced cancer stage at diagnosis (stage III most frequently, but also stage II), luminal B molecular subtype, upper outer quadrant localization of the primary BC tumor, mastectomy, and use of aromatase inhibitors.

Most cases of BC recurrence appear to be age-dependent and correlated with an advanced stage at diagnosis, luminal B molecular subtype, mastectomy, treatment with aromatase inhibitors, and upper-outer quadrant localization of the primary BC tumor. Single-organ regional recurrence was the most common type of recurrence in this patient series. Comprehensive studies on recurrence in postmenopausal women can provide a more precise understanding of the extent of the disease in such patients.

## Figures and Tables

**Table 1:** Demographic and clinical data of the study groups.

	Overall (*n* = 744)
Age of diagnostic	
Mean (SD)	62.4 (9.2)
Range	33.0–87.0
Gender	
Female	736 (98.9%)
Male	8 (1.1%)
Provenience	
Rural	147 (19.8%)
Urban	597 (80.2%)
Stage of cancer	
I	81 (10.9%)
I A	8 (1.1%)
I C	4 (0.5%)
II	5 (0.7%)
II A	171 (23.0%)
II B	125 (16.8%)
III	10 (1.3%)
III A	88 (11.8%)
III B	114 (15.3%)
III C	26 (3.5%)
IV	112 (15.1%)
Immunotherapy	
No	632 (84.9%)
Yes	112 (15.1%)
Radiotherapy	
No	334 (44.9%)
Yes	410 (55.1%)
Chemotherapy	
No	123 (16.5%)
Yes	621 (83.5%)
Hormonotherapy	
No	149 (20.0%)
Yes	595 (80.0%)
Type of recurrence	
N-Miss	614
Distant recurrence	3 (2.3%)
Local	25 (19.2%)
Multiorgan	35 (26.9%)
Single organ	67 (51.5%)

**Table 2:** Distribution of Recurrence Types by Stage of Disease in Patients with Breast Cancer.

Stage of Disease	Distant Recurrence *n* (%)	Local Recurrence *n* (%)	Multiorgan Recurrence *n* (%)	Single-Organ Recurrence *n* (%)	Total *n* (%)	*p* Value
I	0 (0.0)	0 (0.0)	1 (2.9)	4 (6.0)	5 (3.8)	0.3011 ^1^
II	0 (0.0)	0 (0.0)	0 (0.0)	1 (1.5)	1 (0.8)	
IIA	1 (33.3)	12 (48.0)	4 (11.4)	10 (14.9)	27 (20.8)	
IIB	1 (33.3)	3 (12.0)	5 (14.3)	11 (16.4)	20 (15.4)	
III	0 (0.0)	1 (4.0)	2 (5.7)	0 (0.0)	3 (2.3)	
IIIA	1 (33.3)	4 (16.0)	5 (14.3)	13 (19.4)	23 (17.7)	
IIIB	0 (0.0)	4 (16.0)	11 (31.4)	17 (25.4)	32 (24.6)	
IIIC	0 (0.0)	1 (4.0)	5 (14.3)	5 (7.5)	11 (8.5)	
IV	0 (0.0)	0 (0.0)	2 (5.7)	6 (9.0)	8 (6.2)	

^1^ Pearson’s Chi-squared test.

**Table 3:** Distribution of Recurrence Patterns by Breast Cancer Molecular Subtype.

	A (*n* = 269)	B (*n* = 307)	Luminal Her+ (*n* = 79)	Nonluminal Her+ (*n* = 31)	TNBC (*n* = 58)	Total (*n* = 744)	*p* Value
Type_of_recurrence							0.035 ^1^
N-Miss	231.0	244.0	65.0	28.0	46.0	614.0	
Distant recurrence	1.0 (2.6%)	1.0 (1.6%)	0.0 (0.0%)	1.0 (33.3%)	0.0 (0.0%)	3.0 (2.3%)	
Local	8.0 (21.1%)	9.0 (14.3%)	4.0 (28.6%)	0.0 (0.0%)	4.0 (33.3%)	25.0 (19.2%)	
Multiorgan	13.0 (34.2%)	19.0 (30.2%)	1.0 (7.1%)	0.0 (0.0%)	2.0 (16.7%)	35.0 (26.9%)	
Single organ	16.0 (42.1%)	34.0 (54.0%)	9.0 (64.3%)	2.0 (66.7%)	6.0 (50.0%)	67.0 (51.5%)	

^1^ Pearson’s Chi-squared test.

**Table 4:** Distribution of Recurrence Patterns by Treatment Modality in Patients with Documented Recurrence.

	Distant Recurrence (*n* = 3)	Local (*n* = 25)	Multi Organ (*n* = 35)	Single Organ (*n* = 67)	Total (*n* = 130)	*p* Value
Immunotherapy						0.177 ^1^
No	2.0 (66.7%)	18.0 (72.0%)	32.0 (91.4%)	50.0 (74.6%)	102.0 (78.5%)	
Yes	1.0 (33.3%)	7.0 (28.0%)	3.0 (8.6%)	17.0 (25.4%)	28.0 (21.5%)	
Chemotherapy						0.334 ^1^
No	0.0 (0.0%)	1.0 (4.0%)	6.0 (17.1%)	6.0 (9.0%)	13.0 (10.0%)	
Yes	3.0 (100.0%)	24.0 (96.0%)	29.0 (82.9%)	61.0 (91.0%)	117.0 (90.0%)	
RT						0.861 ^1^
No	1.0 (33.3%)	6.0 (24.0%)	12.0 (34.3%)	20.0 (29.9%)	39.0 (30.0%)	
Yes	2.0 (66.7%)	19.0 (76.0%)	23.0 (65.7%)	47.0 (70.1%)	91.0 (70.0%)	
Hormonotherapy						0.681 ^1^
No	1.0 (33.3%)	5.0 (20.0%)	4.0 (11.4%)	11.0 (16.4%)	21.0 (16.2%)	
Yes	2.0 (66.7%)	20.0 (80.0%)	31.0 (88.6%)	56.0 (83.6%)	109.0 (83.8%)	

^1^ Pearson’s Chi-squared test.

**Table 5:** Distribution of Recurrence Patterns by Primary Tumor Quadrant.

Tumor Localization	Distant Recurrence (*n* = 3)	Local (*n* = 25)	Multiorgan (*n* = 35)	Single Organ (*n* = 67)	Total (*n* = 130)	*p* Value
Tumor localization						0.839 ^1^
CS	0.0 (0.0%)	3.0 (12.0%)	9.0 (25.7%)	12.0 (17.9%)	24.0 (18.5%)	
LOQ	0.0 (0.0%)	1.0 (4.0%)	2.0 (5.7%)	8.0 (11.9%)	11.0 (8.5%)	
LIQ	0.0 (0.0%)	2.0 (8.0%)	2.0 (5.7%)	3.0 (4.5%)	7.0 (5.4%)	
UOQ	2.0 (66.7%)	16.0 (64.0%)	16.0 (45.7%)	31.0 (46.3%)	65.0 (50.0%)	
UIQ	1.0 (33.3%)	3.0 (12.0%)	6.0 (17.1%)	13.0 (19.4%)	23.0 (17.7%)	
Bilateral	0.0 (0.0%)	0.0 (0.0%)	0.0 (0.0%)	0.0 (0.0%)	0.0 (0.0%)	

^1^ Pearson’s Chi-squared test.

**Table 6:** Distribution of Recurrence Types by Tumor Dimension in Patients with Breast Cancer.

Type of Recurrence	<2 cm (*n* = 25)	>2–≤5 cm (*n* = 87)	>5 cm (*n* = 11)	Total (*n* = 123)	*p* Value
Distant	1 (4.0%)	1 (1.1%)	0 (0.0%)	2 (1.6%)	0.6191
Local	3 (12.0%)	20 (23.0%)	1 (9.1%)	24 (19.5%)	
Multiorgan	9 (36.0%)	21 (24.1%)	3 (27.3%)	33 (26.8%)	
Single organ	12 (48.0%)	45 (51.7%)	7 (63.6%)	64 (52.0%)	

**Table 7:** Distribution of Recurrence Types by Treatment Modality in Patients with Breast Cancer Pearson’s chi-squared test.

	Distant Recurrence (*n* = 3)	Local (*n* = 25)	Multiorgan (*n* = 35)	Single Organ (*n* = 67)	Total (*n* = 130)	*p* Value
**Radiotherapy**						0.861 ^1^
**No**	1.0 (33.3%)	6.0 (24.0%)	12.0 (34.3%)	20.0 (29.9%)	39.0 (30.0%)	
**Yes**	2.0 (66.7%)	19.0 (76.0%)	23.0 (65.7%)	47.0 (70.1%)	91.0 (70.0%)	
**Chemotherapy**						0.334 ^1^
**No**	0.0 (0.0%)	1.0 (4.0%)	6.0 (17.1%)	6.0 (9.0%)	13.0 (10.0%)	
**Yes**	3.0 (100.0%)	24.0 (96.0%)	29.0 (82.9%)	61.0 (91.0%)	117.0 (90.0%)	
**Immunotherapy**						0.177 ^1^
**No**	2.0 (66.7%)	18.0 (72.0%)	32.0 (91.4%)	50.0 (74.6%)	102.0 (78.5%)	
**Yes**	1.0 (33.3%)	7.0 (28.0%)	3.0 (8.6%)	17.0 (25.4%)	28.0 (21.5%)	
**Hormonotherapy**						0.681 ^1^
**No**	1.0 (33.3%)	5.0 (20.0%)	4.0 (11.4%)	11.0 (16.4%)	21.0 (16.2%)	
**Yes**	2.0 (66.7%)	20.0 (80.0%)	31.0 (88.6%)	56.0 (83.6%)	109.0 (83.8%)	

^1^ Pearson’s Chi-squared test.

## Data Availability

The original contributions presented in this study are included in the article. Further inquiries can be directed to the corresponding author.

## Abbreviations

The following abbreviations are used in this manuscript: BC—Breast Cancer, ER—Estrogen Receptor, PR—Progesterone Receptor, BCS—Breast Conserving Surgery, IRB—Institutional Review Board, TNM—Tumor-Nodes-Metastasis, IHC—Immunohistochemistry, SPSS—Statistical Software for Social Sciences, MHT—Menopausal Hormonal Treatment, AJCC—American Joint Committee on Cancer, AI—Aromatase Inhibitor, ASCO—American Association of Oncology, CAP—College of American Pathologists, CISH—Cytokine Inducible SH2 Containing Protein.
